# Dietary glycemic assessment and type of lens opacity in patients with age-related cataract

**DOI:** 10.1186/1758-5996-7-S1-A225

**Published:** 2015-11-11

**Authors:** Clarissa Simon Factum, Nívea Almeida Casé, Raquel Rocha, Juliete Santos Cortez, Emily David Brandão, Igor Barbosa Mendes, Eduardo Ferrari Marback

**Affiliations:** 1 Universidade Federal da Bahia, Salvador, Brazil

## Objective

To investigate dietary carbohydrate intake, glycemic index and glycemic load and type of lens opacity in patients with age-related cataract.

## Materials and methods

This was an exploratory cross-sectional study, carried out at the Outpatient Clinics of Nutrition and Ophthalmology at the Federal University of Bahia, Salvador-Bahia, Brazil. Seventy eight patients, of both genders, with age-related cataract, participated. All patients underwent nutritional, clinical and ophthalmological assessment. Type of lens opacity was determined following Lens Opacity Classification System – LOCS III – criteria. Clinical data regarding fasting glucose, diabetes diagnosis and hypertension were collected from medical records. Participants answered two 24h-dietary recall. Global dietary carbohydrate intake (CHO), glycemic index (GI) and glycemic load (GL) were estimated.

## Results

Most patients had adequate intake of CHO (83.3%), although presenting moderate dietary GI and high dietary GL (62.3% and 52.6%, respectively). No differences were observed in the distribution of these features in relation to the types of lens opacity (p> 0.05). The presence of posterior subcapsular cataract type (PSC) was higher among patients with hyperglycemia (p=0.009) and diabetes (p=0.031).

## Conclusion

Considering the high prevalence of PSC cataract among those with abnormal blood glucose, nutritional attention should be paid to the quality of dietary carbohydrates in this population.

**Figure 1:**
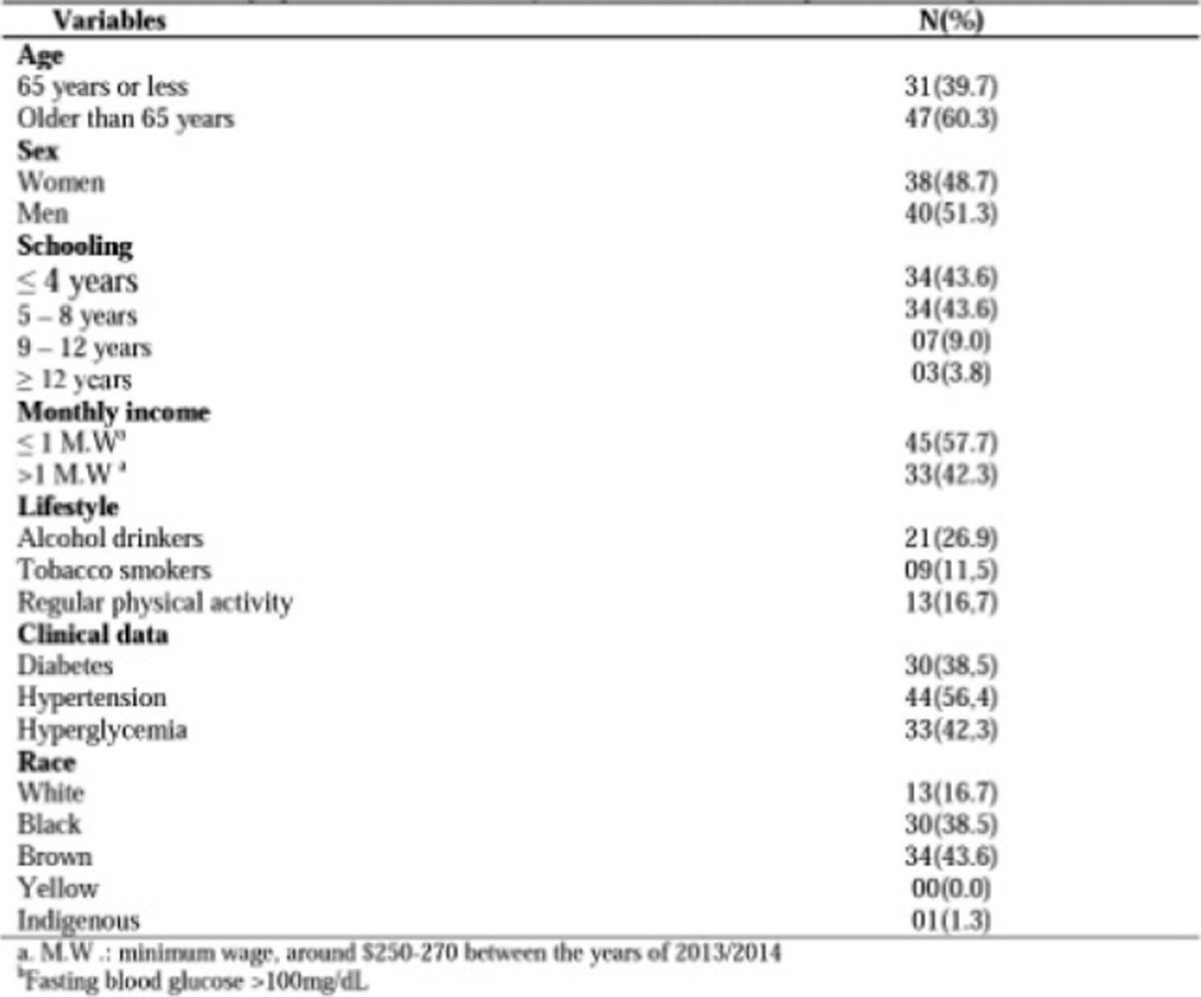
Sociodemographic, clinical and lifestyle characteristics of 78 patients with age-related cataract.

**Figure 2:**
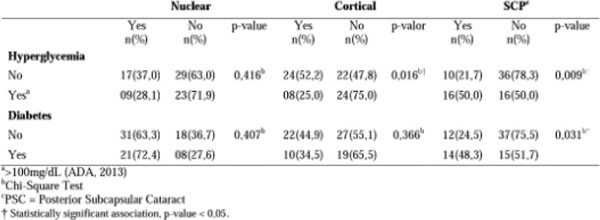
Hyperglycemia and diabetes diagnoses by type of lens opacity in 78 patients with age-related cataract.

**Figure 3:**
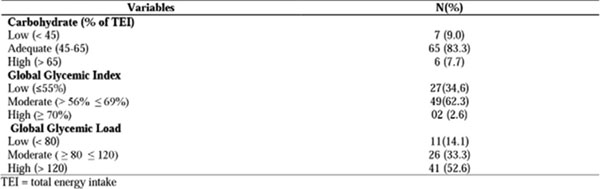
Dietary glycemic assessment of 78 patients with age-related cataract.

**Figure 4:**
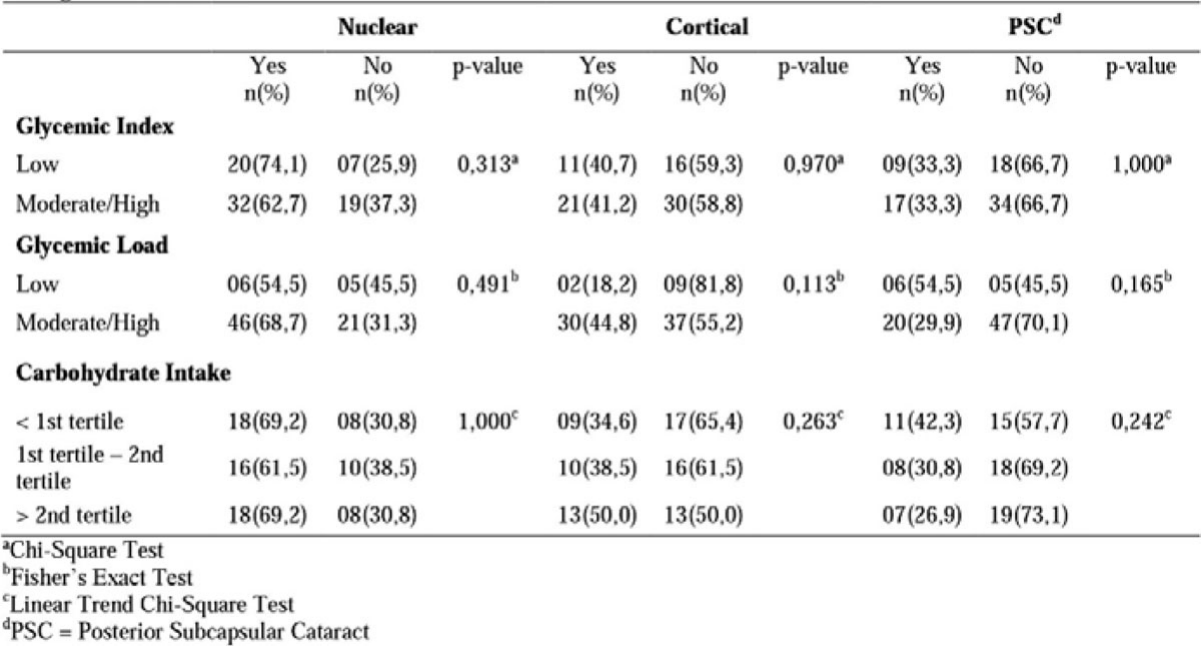
Glycemic Index, glycemic load and total carbohydrate intake among different types of lens opacity in 78 patients with age-related cataract.

